# DPP-4 inhibitors and GLP-1RAs: cardiovascular safety and benefits

**DOI:** 10.1186/s40779-022-00410-2

**Published:** 2022-08-20

**Authors:** Michael Razavi, Ying-Ying Wei, Xiao-Quan Rao, Ji-Xin Zhong

**Affiliations:** 1 Tulane University School of Medicine, New Orleans, LA 70112 USA; 2 Department of Rheumatology and Immunology, Tongji Hospital, Tongji Medical College of Huazhong University of Science and Technology, 1095 Jiefang Ave, Wuhan, 430032 China; 3 Department of Cardiovascular Medicine, Tongji Hospital, Tongji Medical College of Huazhong University of Science and Technology, 1095 Jiefang Ave, Wuhan, 430032 China; 4 Institute of Allergy and Clinical Immunology, Tongji Hospital, Tongji Medical College of Huazhong University of Science and Technology, Wuhan, 430032 China

**Keywords:** Glucagon-like peptide-1 receptor agonists, Dipeptidyl peptidase-4 inhibitors, Type 2 diabetes mellitus, Cardiovascular outcome

## Abstract

Glucagon-like peptide-1 receptor agonists and dipeptidyl peptidase-4 inhibitors are commonly used treatments for patients with type 2 diabetes mellitus (T2DM). Both anti-diabetic treatments function by playing key modulatory roles in the incretin system. Though these drugs have been deemed effective in treating T2DM, the Food and Drug Administration (FDA) and some members of the scientific community have questioned the safety of these therapeutics relative to important cardiovascular endpoints. As a result, since 2008, the FDA has required all new drugs for glycemic control in T2DM patients to demonstrate cardiovascular safety. The present review article strives to assess the safety and benefits of incretin-based therapy, a new class of antidiabetic drug, on the health of patient cardiovascular systems. In the process, this review will also provide a physiological overview of the incretin system and how key components function in T2DM.

## Background

Type 2 diabetes mellitus (T2DM), a common metabolic disorder characterized by insulin resistance and inadequate insulin secretion by pancreatic β cells, has become a growing cause of morbidity and mortality worldwide. The mortality risk associated with T2DM is only secondary to an increase in an individual’s risk of acquiring other disease states that are both vascular and nonvascular in origin [1]. Modifiable risk factors, such as diet and exercise, are important targets for intervention in the management of T2DM [2, 3]. However, for many patients, lifestyle modification alone is not adequate to prevent further disease progression and new disease onset. Several trials, such as the United Kingdom Prospective Diabetes Study (UKPDS) and Action in Diabetes and Vascular Disease: Preterax and Diamicron MR Controlled Evaluation (ADVANCE), have demonstrated that appropriate control of blood glucose is beneficial for the reduction of long-term cardiovascular complications [4, 5]. Although diabetes is widely accepted as one of the major risk factors for cardiovascular disease, the cardiovascular safety of antidiabetic drugs has been questioned due to the finding that intensive glycemic control by antidiabetic medications may result in an unexpected increase in cardiovascular death under certain circumstances [6]. Also, the adverse cardiovascular safety in clinical trials before 2008 was evaluated based on populations that were not necessarily at high risk of cardiovascular disease and the follow-up duration was relatively short (usually less than 12 months). Therefore, the Food and Drug Administration (FDA) released a guidance requiring all new drugs for glycemic control in T2DM patients to demonstrate cardiovascular safety, a policy that was continued and further emphasized in the 2020 FDA Guidance for Diabetes Drug Development [7, 8]. Pharmacological treatment modalities such as glucagon-like peptide-1 (GLP-1) receptor agonists and dipeptidyl peptidase-4 (DPP-4) inhibitors have become increasingly accepted as viable antidiabetic treatment options due to their safety and favorable cardiovascular profiles [9, 10]. There has been a rapid increase in information from large-scale clinical trials assessing the cardiovascular safety and efficacy of incretin-based therapies since 2008. Therefore, an updated critical review of the cardiovascular actions of these therapies, especially information from recently completed trials is needed. The aim of this review was to summarize the recent updates from both clinical trials and preclinical experiments on the impact of GLP-1 receptor agonists (GLP-1RAs) and DPP-4 inhibitors (DPP-4i) on cardiovascular outcomes.

## Molecular bases of incretin system

### Synthesis and distribution of GLP-1

GLP-1, GLP-2, and glucose-dependent insulinotropic polypeptide (GIP) are the predominant gut-derived incretin hormones that lead to postprandial insulin secretion in a glucose-dependent fashion. GLP-1 was originally identified as a 37 amino acid (GLP-1_1–37_) long peptide. However, subsequent studies identified that biologically active GLP-1 is cleaved to contain only 31 amino acids (GLP-1_7–37_). It is also known that there is an isoform of GLP-1, which is one amino acid shorter in both the full length (GLP-1_1–36_) and its active form (GLP-1_7–36_). The biologically active forms of GLP-1 are rapidly degraded by DPP-4, resulting in the production of inactive forms of GLP-1 (GLP-1_9–37_ and GLP-1_9–36_) [11].

GLP-1 is primarily produced by enteroendocrine L-cells that are dispersed throughout the small and large intestines. Yet, GLP-1 is mainly localized to the distal small bowel and colon and is released following nutrient intake [12, 13] and it has been shown that M1 and M2 muscarinic receptors may also be involved in the regulation of GLP-1 release [14].

### Molecular structure of DPP-4

**Fig. 1:**
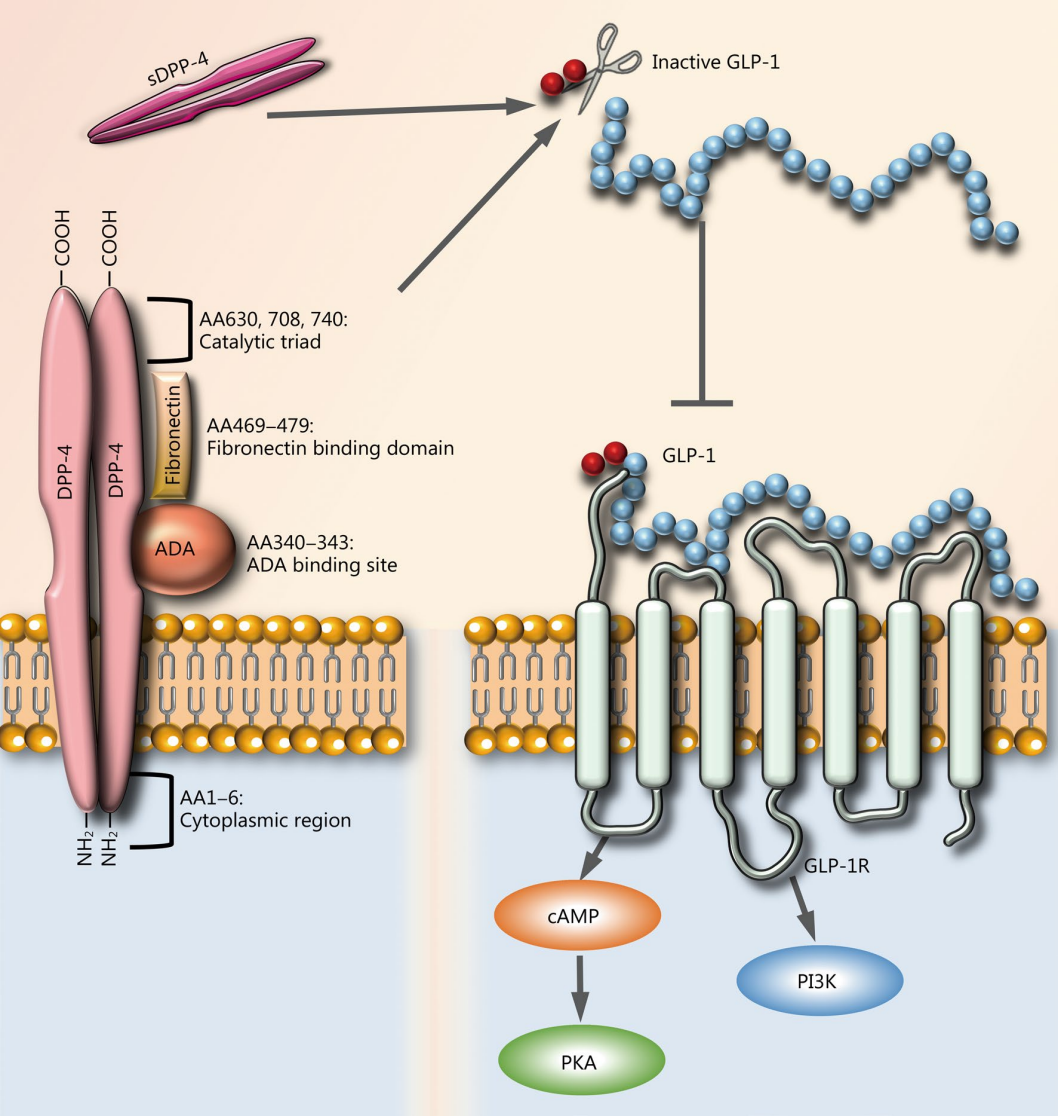
Molecular basis of incretin axis: DPP-4 proteins consist of a short intracellular domain (6 amino acids), a transmembrane domain, and a large extracellular domain. The extracellular domain is responsible for the enzymatic cleavage of the substrates and binding to its ligands including fibronectin and ADA. DPP-4 inactivates GLP-1 by removing N-terminal dipeptide His7Ala8 from active form of GLP-1, which results in the loss of its affinity to GLP-1R. GLP-1R is a G-protein coupled receptor and its biding with active GLP-1 activates PI3K and PKA pathway by increasing intracellular cAMP concentration. DPP-4 dipeptidyl peptidase-4, sDPP-4 soluble DPP-4, AA amino acid, ADA adenosine deaminase, GLP-1 glucagon-like peptide-1, GLP-1R glucagon-like peptide-1 receptor, cAMP cyclic adenosine monophosphate, PKA protein kinase A, PI3K phosphoinositide 3-kinase

DPP-4 has two domains and two subdomains: α/β hydrolase domain (Gln508-Pro766), β-propeller domain (Arg54-Asn497), receptor binding subdomain, and a core subdomain [15]. DPP-4 was first identified as CD26, and is a 110 kD protein that can be found on the cell surface as a monomer, homodimer, or homotetramer. The homodimer form of DPP-4 is the main catalytically active form. The proteolytically active DPP-4 homodimer is found in two forms: a single-pass type II integral transmembrane protein and a soluble protein stripped of any membrane spanning regions or intracellular regions [16]. DPP-4 carries out its exopeptidase activity by cleaving proteins or peptides after encountering a proline or alanine in the second position from the N-terminal end of the amino acid chain. Residues 630, 708, and 740 form the catalytic triad and are indispensable for its catalytic activity. Other functionally crucial residues are 294 and 340–343 within the cysteine-rich domain, which function as adenosine deaminase (ADA) binding domains [16]. Therefore, the functional domains responsible for currently discovered function of DPP-4 are all located in the extracellular portion and the functions of intracellular domain of DPP-4 remain elusive. The structures of DPP-4 and GLP-1 are illustrated in Fig. 1.

## Function and regulation—incretin axis

### GLP-1 and its receptor

GLP-1 secretion, in response to nutrient intake, leads to the release of insulin through the GLP-1 receptor (GLP-1R) and together with other incretin hormones accounts for 50–70% of total post-prandial insulin release [17].

The GLP-1R is a G-protein coupled receptor that has wide tissue expression, including in the brain, the wall of the portal vein near the hilus of the liver, and arteries and arterioles from various organs (kidney, heart, pancreas, and intestine) [18]. The expression of GLP-1 has been seen to spike following gastric bypass surgery and this increase in GLP-1 levels is thought to play a role in appetite suppression and is responsible for postsurgical weight loss and improvements in glucose metabolism [19]. Additionally, agonists of GLP-1R have been associated with reduced fasting very low-density lipoprotein (VLDL), which was shown to be weight-independent [20, 21]. A recent large-scale clinical trial has confirmed that tirzepatide, a dual GIP and GLP-1RAs, resulted in a weight loss of up to 20.9% in adults with body fat mass of 30 or more after 72-week follow-up [22].

Outside of these functions, GLP receptors are also expressed in the human retina and are thought to be involved in the prevention of retinal neurodegeneration, an early event in diabetic retinopathy [23]. Additionally, both GLP-1 and GIP play a role in cardiovascular disease, as agonists of GLP-1 have been shown to reduce platelet activation, thus supporting a protective effect of GLP-1 against microvascular thrombosis [24]. Furthermore, GLP-1 plays a protective role in cardiac microvasculature in diabetes by preventing apoptosis, oxidative stress, and microvascular dysfunction. GLP-1 is thought to mediate this through inhibiting Rho GTPase in the cyclic adenosine 3′,5′-monophosphate (cAMP)/protein kinase A (PKA) pathway [25]. Although the effects of GLP-1 are mostly consistent with an anti-atherogenic function, GIP may have opposing effects that may be viewed as pro-atherogenic. For example, in short term infusion studies in humans, GIP increases the level of osteopontin, which is considered to be a pro-atherogenic cytokine [26].

Despite the wide presence of GLP-1R throughout bodily tissues, it is predominantly found in islet β-cells and postprandial insulin release from these cells is dependent upon the ability of GLP-1 to interact with GLP-1R [27]. This process follows a known pathway in which the interaction between GLP-1 and its receptor, initiates Gαs-protein coupling. This leads to the immediate release of cAMP and the eventual downstream release of calcium and β-arrestin. The downstream release of these three factors, in response to GLP-1R activation, leads to glucose-dependent insulin release. Studies in which GLP-1R was deleted or knocked out in murine models resulted in impaired glucose-dependent insulin release. Thus, GLP-1R is thought to be a key component of the incretin effect—insulin secretion in response to oral glucose intake [27].

### Regulation of incretin system

Current literature has shown that somatostatin can lower levels of GLP-1 released during the fasting state, suggesting the existence of basal levels of GLP [28]. There is an approximately threefold increase in GLP from basal levels, observed in response to oral intake of glucose [28]. To this end, GLP-1 is not produced in response to intravenous glucose and instead, the amount of GLP-1 released is relative to the size of a meal and is correlated to the rate of gastric emptying [29]. It has also been shown that the response of GLP-1 to meals is unaffected by small intestinal resections which can interrupt intramural reflux pathways [30].

Following their release into the blood stream, incretin peptides are rapidly inactivated by enzymatic cleavage. DPP-4 is a key modulator of the incretin system, and functions to catalytically inactivate GLP-1 and GIP. Specifically, GLP-1 and GIP are cleaved into two biologically inactive forms, GLP-1_9–37_ (or GLP-1_9–36_) and GIP_3–42_. Rapid inactivation of these incretin hormones by DPP-4 leads to an insufficient release of insulin following oral glucose intake [31] and many of the physiological signs of T2DM, such as deteriorating glycemic control, are secondary to incretin inactivation by DPP-4. DPP-4 expression is positively correlated with glycated hemoglobin (HbA1c), adipocyte size, inflammation, and visceral adipose tissue [17, 32].

Overall, regulation of DPP-4 remains nebulous. However, previous studies have found that DPP-4 can be regulated by inflammatory factors such as signal transducer and activator of transcription 1α (STAT1α), hepatocyte nuclear factor-1α (HNF-1α), interleukin-12 (IL-12), and tumor necrosis factor-α (TNF-α) [33–35]. Specifically, STAT1α is thought to play a role in transcriptional regulation as the promoter region of DPP-4 contains an interferon-gamma-activated sequence (GAS), which is an activated STAT1α binding site. STAT1α becomes active in response to retinoic acid and interferon α, β, and γ, leading to the binding of STAT1α to GAS in the DPP-4 promoter. This mechanism of promoter binding leads to increased DPP-4 transcription [33]. Similarly, T-helper cell 17 skewing condition (TGF-β, IL-23, IL-6, IL-1β, and IL-21) also results in increased DPP-4 expression [34]. Furthermore, IL-12 is thought to promote DPP-4 translation, whereas TNF-α is thought to decrease its expression [34, 35]. Our recent work demonstrated that oxidized low-density lipoprotein (LDL) upregulates DPP-4 expression on macrophages via activation of Toll-like receptor 4 (TLR4)/TIR-domain-containing adapter-inducing interferon-β (TRIF) and CD36 pathways [36]. Decreased DPP-4 or CD26 expression is also seen in cases where CD9 is deleted and in murine models of hyperoxygenation [35]. Related to relative oxygen concentration, DPP-4 is thought to be released [potentially mediated by matrix metalloproteases (MMPs)] in human smooth muscle cells under hypoxic conditions. To this end, HIF-1α and HNFs have been shown to target DPP-4 expression [35]. Hypoxic conditions can also lead to a phenomenon known as DPP-4 shedding whereby transmembrane DPP-4 is cleaved from the membrane and released into the circulation in a soluble form. Hypoxia-induced shedding is thought to occur due to an intricate interaction between various MMPs [37].

**Fig. 2:**
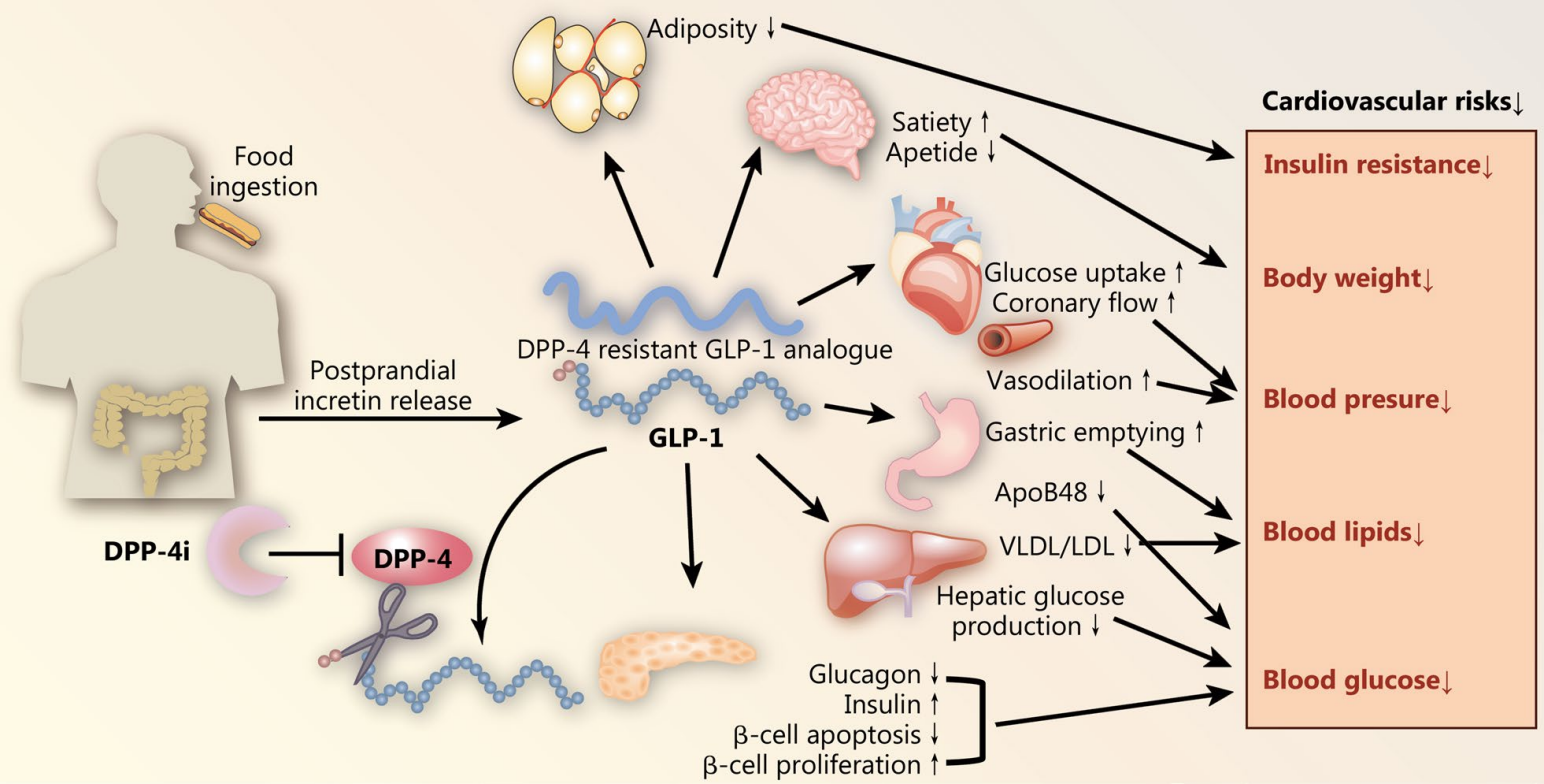
Incretin axis and incretin-based therapies: GLP-1 is produced by the enteroendocrine L-cells in response to meal ingestion. The active form of GLP-1 is rapidly inactivated by DPP-4. GLP-1 acts on pancreas, liver, gastrointestinal tract, adipose tissue, cardiovascular system, and brain to exert a variety of functions. The rapid inactivation by DPP-4 in vivo limits the application of GLP-1 in clinic. The development of DPP-4 resistant analogues (GLP-1RAs) and DPP-4i overcame the instability shortcoming of GLP-1 and became an important class of glycemic lowering drugs that are safe or beneficial to cardiovascular disease. GLP-1RAs reduce multiple cardiovascular risks such as hypertension, hyperglycemia, dyslipidemia, overweight, and insulin resistance via various mechanisms. DPP-4 dipeptidyl peptidase-4, DPP-4i DPP-4 inhibitors, GLP-1 glucagon-like peptide-1, ApoB48 apolipoprotein B48, VLDL very low-density lipoprotein, LDL low-density lipoprotein

In summary, GLP-1 and its analogues appear to exert multiple important actions in brain, liver, muscle and fat in addition to its main action of stimulating insulin secretion. However, the rapid inactivation of GLP-1 by DPP-4 in vivo limits its application in clinic. By preserving GLP-1, DPP-4i enhances GLP-1-induced activities in these tissues (Fig. 2).

## Effects on glycemic control

### Blood glucose lowering effect of GLP-1RAs

As previously noted, DPP-4 rapidly cleaves and inactivates GLP-1, resulting in its very short half-life (2–3 min). While DPP-4 expression is significantly increased in patients with obesity and T2DM, the incretin effect is markedly impaired. Early studies revealed that GLP-1 injections improved postprandial insulin release, lowered fasting glucose and HbA1c levels, and led to modest weight loss [38]. Exendin-4 (exenatide), a GLP-1 peptide encoded by lizard genes that is resistant to DPP-4-mediated catalytic degradation, received FDA approval in 2005 and became the first GLP-1RA available for public use. Between 2005 and 2021, the following GLP-1 agonists were also approved by the FDA for parenteral use: liraglutide, albiglutide, lixisenatide, dulaglutide, and semaglutide. Lixisenatide was also approved during that timeframe, for use in Europe [31]. The individual agents differ in their unique pharmacology such as half-life and degree of structural homology to GLP-1 [39]. As a class, however, these agents work similarly where they mimic the structure of GLP-1 in vivo*,* and thus, can bind the GLP-1R, but are not subject to degradation by DPP-4. This degradation by DPP-4 is evaded because most GLP-1 analogues contain an amino acid substitution at the N-terminal end. For example, exendin-based GLP-1 analogues have an alanine to glutamic acid substitution in the second position [40].

Overall, GLP-1RAs have been shown to be effective in managing T2DM and have helped patients reduce HbA1c by 2% over the course of 18 months to reach levels below 7% [41]. Likewise, they can increase insulin secretion by improving beta-cell survival and suppressing glucagon secretion by improving alpha-cell health and glucose sensing ability. Additionally, GLP-1RAs were able to address the pathophysiology of T2DM through an indirect mechanism also. Slowed gastric emptying, weight loss, and favorable results on lipid parameters have all been associated with GLP-1RAs use [40]. The overall mechanisms by which GLP-1RAs carry out their cardiometabolic effects are still unclear, however recent studies suggest that this process may be mediated by reduction of inflammation and epicardial adipose tissue [42, 43].

### Blood glucose lowering effect of DPP-4i

DPP-4i are oral anti-diabetic drugs that function by blocking incretin degradation caused by DPP-4, as outlined earlier in this review. Blocking the catalytic breakdown of GLP-1 and GIP, allows for postprandial insulin release [44]. Murine-based studies have shown that DPP-4 inhibition directly results in increased GLP-1 secretion and improves both overall insulin section and glucose tolerance. To this end, DPP-4 knockout mice also showed similar findings, indicating that DPP-4 inhibition is an effective mechanism for treating T2DM [45].

The FDA approved sitagliptin, the first DPP-4i on the market, in 2006 and shortly after, numerous additional DPP-4i received market clearance. The FDA approved saxagliptin, linagliptin, and alogliptin, and the European Union and Japan approved vildagliptin, anagliptin, teneligliptin, trelagliptin, and omarigliptin. Gemigliptin and evogliptin were soon approved in Korea, and gosogliptin was approved in Russia. These inhibitors differ based on which of the two DPP-4 structural classes they belong to (non-peptidomimetics or dipeptide structural mimics), their metabolism (liver vs. kidney routes), their excretion, their elimination half-life, their target selectivity, and their effective dosage [46].

Collective data from major clinical trials have shown that treatment with a DPP-4i improves glycemic control by increasing insulin secretion from islet cells, lowering levels of HbA1c, reducing adipocyte size, and suppressing inflammation [47–51]. Unlike GLP-1RAs, DPP-4i have no effect on gastric emptying or weight loss. In addition to improved glycemic control, Nakamura et al. [52] showed that a 12-month treatment with sitagliptin significantly decreased systolic [( − 7.0 ± 18.9) mmHg] and diastolic [( − 5.1 ± 11.7) mmHg] blood pressure in patients with both T2DM and cardiovascular risk factors. A slight reduction in blood pressure has also been observed in SUSTAIN-6 and LEADER trials [53, 54]. However, this effect was not seen in other major cardiovascular outcome trials (CVOTs) and their blood pressure lowering effect remains controversial, which will be further discussed below.

## CVOTs

In response to FDA guidelines requiring the assessment of anti-diabetic therapeutics in relation to cardiovascular risk, several CVOTs have been performed. These outcome trials aimed to determine cardiovascular safety and thus most of them were designed to show non-inferiority of the drug against placebo. To rule out inferiority, the FDA required CVOTs to show an upper boundary risk ratio of 1.3 (post approval), utilizing a two-sided 95% confidence interval (CI), for major adverse cardiovascular events (MACE) [55]. In this section, we reviewed all major CVOTs with published results by searching the MEDLINE and EMBASE databases using the following terms: 1) glucagon-like peptide-1 agonists OR GLP-1RAs; 2) glucagon like peptide OR GLP-1; 3) exenatide OR lixisenatide OR liraglutide OR semaglutide OR albiglutide OR dulaglutide OR efpeglenatide; 4) randomized controlled trial; 5) cardiovascular outcome OR cardiovascular safety OR cardiovascular events; 6) DPP4 OR DPP-4 OR DPP-IV; 7) dipeptidyl peptidase-4; and 8) alogliptin OR saxagliptin OR sitagliptin OR linagliptin OR vildagliptin. The search strategy [(1 OR 2 OR 3) AND 4 AND 5] was used for CVOTs with GLP-1RAs and [(6 OR 7 OR 8) AND 4 AND 5] was used for CVOTs with DPP-4i. Trials with less than 4000 patient-years of exposure to the drug were excluded according to the updated FDA Guidance for Diabetes Drug Development released in 2020 [8]. The following CVOTs, relative to DPP-4i and GLP-1RAs, have been identified and included in this review: EXAMINE, SAVOR-TIMI53, TECOS, CARMELINA, CAROLINA, ELIXA, SUSTAIN 6, EXSCEL, HARMONY, LEADER, PIONEER 6, REWIND, and AMPLITUDE-O [47–49, 53, 54, 56–63]. All trials revealed cardiovascular safety by establishing non-inferiority. Interestingly, the superiority analyses showed heterogeneous results among these trials. One important reason for this phenomenon may be the heterogeneity in subject disease conditions, follow-up durations, and kinetics of drugs. In the following sections, we will review these CVOTs on incretin-based therapies in detail.

## Effects on classic cardiovascular risk factors

### CVOTs on GLP-1RAs

Data from the major CVOTs ELIXA, SUSTAIN-6, EXSCEL, HARMONY, PIONEER 6, LEADER, REWIND, and AMPLITUDE-O [53, 54, 56, 59–63] support the overall safety of GLP-1RAs relative to major cardiovascular outcomes. Specifically, the ELIXA (lixisenatide) trial studied patients with T2DM who had recent acute coronary syndrome and found that the addition of lixisenatide to conventional therapy did not result in a significant difference in cardiovascular endpoints relative to placebo (Table 1) [56]. SUSTAIN-6 (semaglutide) [53], PIONEER 6 (semaglutide) [61], and LEADER (liraglutide) [54] looked at similar patient populations (over 50 years old with established cardiovascular disease) and found that GLP-1RAs produce a cardiovascular protective function relative to primary endpoints (Table 1). Both SUSTAIN-6 and LEADER showed a statistically significant decrease in MACE [53, 54]. Although PIONEER 6 only showed an insignificant reduction of MACE, subcategory analysis demonstrated significant improvement in all-cause mortality [1.4% vs. 2.8%, hazard ratio (*HR*) = 0.51, 95% CI 0.31–0.84] and cardiovascular mortality (0.9% vs. 1.9%, *HR* = 0.49, 95% CI 0.27–0.92) [61]. The HARMONY trial studied T2DM patients, 40 years and older, with established cardiovascular disease and reported that albiglutide reduced the primary composite outcome. The incidence rates of the primary composite outcome were 4.6 and 5.9 events per 100 person-years for albiglutide vs. placebo respectively (*HR* = 0.78, 95% CI 0.68–0.90, *P* < 0.0001 for non-inferiority; *P* = 0.0006 for superiority) [60]. Furthermore, Ferdinand et al. [64] found that dulaglutide, a long-lasting form of GLP-1RA administrated once weekly, is also safe and does not increase the risk of major cardiovascular events in patients with T2DM. The recently completed large-scale REWIND trial randomized 9901 patients with T2DM to either placebo or dulaglutide. Unlike its predecessors, the REWIND trial was not aimed at uncovering cardiovascular safety of GLP-1RAs. Rather, it focused on specifically determining the cardiovascular superiority of dulaglutide relative to cardiovascular endpoints. During a median follow-up of 5.4 (IQR 5.1–5.9) years, patients with a 1.5 mg/week subcutaneous injection of dulaglutide showed a lower incidence rate of the primary composite outcome including non-fatal myocardial infarction, non-fatal stroke, or death from cardiovascular causes (*HR* = 0.88, 95% CI 0.79–0.99, *P* = 0.026) [62]. Additionally, compared to glimepiride, a commonly used sulfonylurea, exenatide was able to significantly reduce cardiovascular risk factors such as body mass index, blood pressure, and high-density lipoprotein (HDL). These results suggest that GLP-1RAs may be preferred to sulfonylureas as an add-on therapy [65]. Efpeglenatide, an exendin 4-based molecule, also showed a significant reduction in major adverse cardiovascular events (7.0% vs. 9.2%, *HR* = 0.73, 95%CI 0.58–0.92, *P* < 0.001 for non-inferiority; *P* = 0.007 for superiority) in T2DM patients with a history of cardiovascular disease or current kidney disease in a recently completed trial (AMPLITUDE-O) [63]. This result suggested that the long-acting form, unlike the short-acting forms (lixisenatide and exenatide), of exendin-4-based GLP-1RAs improved cardiovascular outcomes. This also supports the finding that the cardiovascular benefits are not restricted to those agents structurally similar to human GLP-1 but are more likely a class effect that is seen in all GLP-1RAs. As a class, GLP-1RAs were also seen to reduce all-cause mortality by 11% compared to placebo [54]. Specific to cardiovascular-related death, ELIXA and SUSTAIN-6 trials showed similar performance relative to the placebo [*HR* = 0.98 (95% CI 0.78–1.22) and *HR* = 0.98 (95% CI 0.65–1.48), respectively]. Conversely, the use of liraglutide in the LEADER trial led to a 22% reduction in cardiovascular deaths [53, 54, 56].

**Table 1:** Summary of main CVOTs for GLP-1RAs

Characteristic	ELIXA (2015) [56]	SUSTAIN-6 (2016) [53]	LEADER (2016) [54]	EXSCEL (2017) [59]	HARMONY (2018) [60]	PIONEER 6 (2019) [61]	REWIND (2019) [62]	AMPLITUDE-O (2021) [63]
Drug	Lixisenatide	Semaglutide	Liraglutide	Exenatide	Albiglutide	Semaglutide	Dulaglutide	Efpeglenatide
Control	Placebo	Placebo	Placebo	Placebo	Placebo	Placebo	Placebo	Placebo
Median follow-up (years)	2.1	2.1	3.5	3.2	1.6	1.3	5.4	1.81
Inclusion criteria	T2DM and acute coronary events within the past 180 d	≥ 50 years of age with established CVD, HF (class I or II), CKD (stage 3 +), or ≥ 60 with cardiovascular risk factor	≥ 50 years of age with established CVD, HF (class I or II), CKD (stage 3 +), or ≥ 60 with cardiovascular risk factor	T2DM on 0–3 oral or insulin antidiabetic drugs; HbA1c 6.5–10.0%	≥ 40 years of age with T2DM; HbA1c > 7.0%; established CVD	≥ 50 years; T2DM with established CVD; or ≥ 60 with cardiovascular risk factor	≥ 50 years; T2DM with HbA1c ≤ 9.5%; two or less oral glucose-lowering drugs	T2DM with history of CVD or eGFR 25.0 to 59.9 ml/(min‧1.73 m^2^)
Exclusion criteria	< 30 years of age, PCI within past 15 d, CABG for the qualifying event, planned revascularization, eGFR < 30 ml/(min‧1.73 m^2^), HbA1c < 5.5% or > 11.0%	Treatment with DPP-4i, GLP-1R, or insulin other than basal within 30 d; premixed insulin treatment within 90 d; acute coronary or cerebrovascular event within 90 d; planned coronary intervention	T1DM; use of GLP-1RAs, DPP-4i, or rapid-acting insulin; familial history of medullary thyroid cancer or type 2 endocrine neoplasia; acute coronary or cerebrovascular event within 14 d	T1DM; use of GLP-1RAs; history of gastroparesis, pancreatitis; pregnancy; eGFR < 30 ml/(min‧1.73 m^2^); planned revascularization	eGFR < 30 ml/(min‧1.73 m^2^); use of GLP-1RAs; severe gastroparesis; history of pancreatitis, pancreatic tumors, medullary carcinoma of the thyroid; pregnancy, breastfeeding	Use of DPP-4i, GLP-1R; malignant neoplasms; pancreatitis; HF class IV; planned revascularisation; eGFR < 30 ml/(min‧1.73 m^2^)	eGFR < 15 ml/(min‧1.73 m^2^); cancer; severe hypoglycemia history; life expectancy < 1 year; coronary or cerebrovascular event within 2 months; planned revascularization	History of GI disease, pancreatitis; hypertension; personal or family history of medullary thyroid cancer; planned coronary procedure; use of DPP-4i, GLP-1R; retinopathy or maculopathy
Results of primary endpoint (vs. placebo)	Non-inferiority; *HR* = 1.02 (95% CI 0.89–1.17)	6.6% vs. 8.9%; *HR* = 0.74 (95% CI 0.58–0.95); *P* < 0.001 for non-inferiority; *P* = 0.02 for superiority	*HR* = 0.87 (95% CI 0.78–0.97); *P* < 0.001 for non-inferiority; *P* = 0.01 for superiority	11.4% vs. 12.2%; *HR* = 0.91 (95% CI 0.83–1.00); *P* = 0.06 for superiority	7% vs. 9%; *HR* = 0.78 (95% CI 0.68–0.90); *P* < 0.0001 for non-inferiority; *P* = 0.0006 for superiority	3.8% vs. 4.8%; *HR* = 0.79 (95% CI 0.57–1.11); *P* = 0.17^*^	12.0% vs. 13.4%; *HR* = 0.88 (95% CI 0.79–0.99); *P* = 0.026 for superiority	7.0% vs. 9.2%; *HR* = 0.73 (95% CI 0.58–0.92); *P* < 0.001 for non-inferiority; *P* = 0.007 for superiority
HF	*HR* = 0.96 (95% CI 0.75–1.23)	*HR* = 1.11 (95% CI 0.77–1.61)	4.7% vs. 5.3%; *HR* = 0.87 (95% CI 0.73–1.05)	*HR* = 0.94 (95% CI 0.78–1.13)	*HR* = 0.85 (95% CI 0.70–1.04)	1.3% vs. 1.5%; *HR* = 0.86 (95% CI 0.48–1.55)	*HR* = 0.93 (95% CI 0.77–1.12)	*HR* = 0.61 (95% CI 0.38–0.98)
Myocardial infarction	*HR* = 1.03 (95% CI 0.87–1.22)	*HR* = 0.74 (95% CI 0.51–1.08)	6.3% vs. 7.3%; *HR* = 0.86 (95% CI 0.73–1.00)	*HR* = 0.97 (95% CI 0.85–1.10)	*HR* = 0.75 (95% CI 0.61–0.90)	*HR* = 1.18 (95% CI 0.73–1.90)	4.1% vs. 4.3%; *P* = 0.65	*HR* = 0.75 (95% CI 0.54–1.05)

*Significant improvement in all-cause mortality (1.4% vs. 2.8%, *HR* = 0.51; 95% CI 0.31–0.84), cardiovascular mortality (0.9% vs. 1.9%, *HR* = 0.49; 95% CI 0.27–0.92). *CVOTs* cardiovascular outcome trials, *T2DM* type 2 diabetes mellitus, *CVD* cardiovascular disease, *HF* heart failure, *CKD* chronic kidney disease, *HbA1c* glycated hemoglobin, *eGFR* estimated glomerular filtration rate, *PCI* percutaneous coronary intervention, *CABG* coronary artery bypass graft, *GLP-1R* GLP-1 receptor, *T1DM* type 1 diabetes mellitus, *GLP-1RAs* glucagon-like peptide-1 receptor agonists, *DPP-4i* DPP-4 inhibitor, *HR* hazard ratio, *CI* confidence interval

Except for ELIXA and EXSCEL [56, 59], all other major CVOTs point towards cardiovascular benefits of GLP-1RAs [53, 54, 61, 63, 66–68]. Recent meta-analyses of the major CVOTs for GLP-1RAs have also demonstrated an overall protective effect of GLP-1RAs on cardiovascular events [69, 70]. Evidently, the differences amongst the results point towards in-class heterogeneity. It is noteworthy that the study populations were highly specific in these trials (refer to inclusion and exclusion criteria sections of Table 1) and most trials recruited patients with established cardiovascular disease or having high risk of cardiovascular disease. Therefore, the heterogeneities in cardiovascular medication, baseline characteristics, study population, and study design should be taken into consideration when comparing the results among these trials. Particularly, the background anti-diabetic agents, lipid-lowering drugs, antiaggregants, and antihypertensives may affect cardiovascular events. In addition to the different study populations and study design in these trials, one potential reason for this heterogeneity in cardiovascular benefit is the structural differences among the GLP-1RAs used in these trials. Lixisenatide and exenatide used in ELIXA and EXSCEL trials are short-acting GLP-1RAs that are structurally similar to exendin-4, a peptide found in the saliva of the Gila monster *Heloderma suspectum*. The GLP-1RAs used in the other trials, including liraglutide, semaglutide, dulaglutide, and albiglutide, are all analogous to human GLP-1. It has recently been reported that human GLP-1 analogues were more effective in reducing the occurrence of major adverse cardiovascular events, myocardial infarctions, and hospitalizations due to cardiovascular causes when compared to exendin-based GLP-1RAs [71]. It should also be noted that ELIXA and REWIND were the only two trials where average baseline HbA1c level was lower than 8%. In addition, the follow-up duration of ELIXA was also relatively short (2.1 years), especially when compared with the follow-up duration of 5.4 years in the REWIND trial [60, 62].

GLP-1RAs have been shown to reduce blood pressure, although the exact mechanism is unknown. It is believed that GLP-1RAs induce vasodilation through action of the proximal tubular cells and stimulating urinary sodium excretion [68, 72]. GLP-1 injections were also shown to improve myocardial tolerance to stress induced ischemia at peak activity and 30 min post-activity [73]. Gaspari et al. [74] used an apoliprotein-E deficient (ApoE^−/−^) mouse model to show the beneficial effects of liraglutide as they relate to atherosclerotic vascular disease. The beneficial effects observed in those patients with early onset, low atherosclerotic burden include an improvement in plaque stability score in the brachiocephalic artery, attenuation of lipid deposits in the aorta, and reduction of weight gain. For high burden atherosclerosis, there was an observed attenuation of endothelial dysfunction in the liraglutide treatment group. GLP-1RAs, specifically liraglutide, have also been evaluated for their effects on cardiovascular biomarkers. The specific biomarkers focused on were adiponectin, leptin, IL-6, TNF-α, plasminogen activator inhibitor-1 (PAI-1), brain natriuretic peptide (BNP), and high-sensitivity C-reactive protein (hs-CRP). Increased levels of PAI-1 and BNP were found post-14-week treatment with liraglutide [66].

### CVOTs on DPP-4i

Data from TECOS, EXAMINE, SAVOR-TIMI53, CARMELINA, and CAROLINA showed that DPP-4i are non-inferior to the placebo or glimepiride relative to primary cardiovascular endpoints [17, 48, 49, 57, 58]. Unlike GLP-1RAs, none of the DPP-4i tested displayed superiority over the placebo in relation to the primary endpoint and the detailed results of these three trials are described in Table 2. It is important to note that these clinical trials were designed as non-inferiority studies. Thus, supporting the cardiovascular safety of these drugs and not necessarily their observed benefits. To make firmer conclusions, additional clinical trials designed around revealing cardiovascular benefits should be conducted. Despite the superior cardiorenal benefits of sodium/glucose cotransporter-2 inhibitors (SGLT-2i) and GLP-1RAs [75, 76], DPP-4i remained a common choice among these three classes of anti-diabetic agents, with more patients using DPP-4i than the other two [77]. In-class differences were noticed especially related to heart failure (HF) and are discussed later in this manuscript. Among these major CVOTs, the CAROLINA study compared the cardiovascular safety of linagliptin with that of glimepiride. Relative to glimepiride (sulfonylurea), DPP-4i were not associated with any increase in cardiovascular risk for patients with T2DM [58]. Although no improvement of cardiovascular outcomes was observed, linagliptin showed a better management of blood glucose with a higher rate of maintaining HbA1c below 7.0% (16.0% vs. 10.2%, *HR* = 1.68, 95% CI 1.44–1.96) and a lower incidence of hypoglycemic adverse events (10.6% vs. 37.7%, *HR* = 0.23, 95% CI 0.21–0.26) [58].

**Table 2:** Summary of main CVOTs for DPP-4i

Characteristic	EXAMINE (2013) [48]	SAVOR-TIMI53 (2015) [49]	TECOS (2015) [47]	CARMELINA (2019) [57]	CAROLINA (2019) [58]
Drug	Alogliptin	Saxagliptin	Sitagliptin	Linagliptin	Linagliptin
Comparative agent	Placebo	Placebo	Placebo	Placebo	Glimepiride
Median follow-up period (years)	1.5	2.1	3	2.2	6.3
Inclusion criteria	≥ 18 years with T2DM who are receiving mono or combination therapy (excluding GLP-1RAs or DPP-4i); HbA1c levels between 6.5 and 11.0%; History of ACS within 15–90 days of screening	T2DM with HbA1c between 6.5 and 12.0% and an established cardiovascular disease or multiple vascular risk factors	≥ 50 years of age with T2DM; HbA1c 6.5 and 8.0% when treated with OAD or insulin; established cardiovascular disease	T2DM with HbA1c of 6.5–10.0%; high cardiovascular risk; high renal risk: eGFR 45–75 ml/(min‧1.73 m^2^) and UACR ≥ 200 mg/g, or eGFR 15–45 ml/(min‧1.73 m^2^)	Age 40–85 years; T2DM with increased cardiovascular risk or established CVD; HbA1c of 6.5–8.5%; BMI ≤ 45 kg/m^2^
Exclusion criteria	T1DM; currently on GLP-1RAs; taken DPP-4i for > 14 d or within past 3 months; unstable cardiovascular disorder; dialysis; severe immunodeficiency	Treatment with incretins in the past 6 months; dialysis; prior renal transplant; or serum creatinine higher than 6.0 mg per deciliter	Prior treatment with GLP-1RAs, DPP-4i, thiazolidinedione within the last 3 months; two or more hypoglycemia episodes in the past 12 months; eGFR < 30 ml/(min‧1.73 m^2^)	T1DM; prior use of GLP-1RAs or DPP-4i; eGFR < 15 ml/(min‧1.73 m^2^) or requiring maintenance dialysis; liver disease; bariatric surgery; nursing or pregnant women	T1DM; insulin therapy; prior use of DPP-4i, GLP-1RAs, or thiazolidinedione; uncontrolled hyperglycemia; liver disease; HF class III or IV
Results of primary endpoint (vs. placebo/control)	*HR* = 0.96 (upper boundary of the one-sided repeated CI 1.16; *P* = 0.32 for superiority; *P* < 0.001 for non-inferiority)	*HR* = 1.00 (95% CI 0.89–1.12; *P* = 0.99 for superiority; *P* < 0.001 for non-inferiority)	*HR* = 0.98 (95% CI 0.88–1.09; *P* < 0.001 for non-inferiority; intention-to-treat analysis: 0.98; 95% CI 0.89–1.08; *P* = 0.65 for superiority)	*HR* = 1.02 (95% CI 0.89–1.17); *P* < 0.001 for non-inferiority	11.8% vs. 12.0%; *HR* = 0.98 (95% CI 0.84–1.14; *P* < 0.001 for non-inferiority; *P* = 0.76 for superiority)
HF hospitalization	*HR* = 1.07 (95% CI 0.79–1.46)	3.5% vs. 2.8%; *HR* = 1.27 (95% CI 1.07–1.51; *P* = 0.007)	3.1% vs. 3.1%; *HR* = 1.00 (95% CI 0.83–1.20); *P* = 0.98	6.0% vs. 6.5%; *HR* = 0.90 (95% CI 0.74–1.08); *P* = 0.26	*HR* = 1.21 (95% CI 0.92–1.59); *P* = 0.18
Myocardial infarction	6.5% vs. 6.9%; *HR* = 1.08 (95% CI 0.88–1.33)	*HR* = 0.95 (95% CI 0.80–1.12); *P* = 0.52	*HR* = 0.95 (95% CI 0.81–1.11); *P* = 0.49	*HR* = 0.78 (95% CI 0.36–1.72); *P* = 0.54^*^	*HR* = 1.03 (95% CI 0.82–1.29); *P* = 0.82
All-cause mortality	6.5% vs. 5.7%; *HR* = 0.88 (95% CI 0.71 – 1.09)	*HR* = 1.11 (95% CI 0.96–1.27); *P* = 0.15	*HR* = 1.01 (95% CI 0.90–1.14); *P* = 0.88	10.5% vs. 10.7%; *HR* = 0.98 (95% CI 0.84–1.13); *P* = 0.74	*HR* = 0.91 (95% CI 0.78–1.06); *P* = 0.23

*Fatal myocardial infarction. *CVOTs* cardiovascular outcome trials, *T2DM* type 2 diabetes mellitus, *GLP-1RAs* GLP-1 receptor agonists, *HbA1c* glycated hemoglobin, *ACS* acute coronary syndrome, *OAD* oral antidiabetic drugs, *eGFR* estimated glomerular filtration rate, *UACR* urine albumin-creatinine ratio, *CVD* cardiovascular disease, *BMI* body mass index, *T1DM* type 1 diabetes mellitus, *DPP-4i* DPP-4 inhibitor, *HR* hazard ratio, *CI* confidence interval, *HF* heart failure

Outside of those major clinical trials, a nationwide retrospective study in Taiwan, China, evaluated the effects of DPP-4i in elderly patients with T2DM relative to cardiovascular outcomes. Specifically, Shih et al. [67] studied a cohort of over 400,000 T2DM patients above the age of 65 and found that DPP-4i were associated with a 21% decrease in the risk of MACEs (including myocardial infarction and ischemic stroke) with a 46% overall decrease in risk of all-cause mortality. Furthermore, in this same cohort, there were no significant differences in the rates of hospitalization for HF between the treatment and placebo groups. These findings were also found consistent across comorbidity subgroups [67].

DPP-4i have been associated with a reduction in atherosclerosis and inflammation in several studies utilizing animal models [78–80]. The effect of sitagliptin on coronary atherosclerosis was studied utilizing intravascular ultrasound (IVUS). A non-significant reduction in coronary plaque volume, decrease in liquid plaque volume, and a decrease in non-HDL cholesterol was observed in the sitagliptin group [81]. Additionally, de Boer et al. [51] found that linagliptin decreases aortic pulse wave velocity (PWV), a surrogate marker for arterial stiffness and early atherosclerosis, by an average of 0.91 m/s.

### Mechanisms underlying cardiovascular effects of incretin therapy

In addition to glycemic effects, GLP-1RAs also favorably modulate multiple cardiovascular risk factors via acting on a variety of organ systems. By signaling through GLP-1R expressed in the reward and satiety areas of central nervous system, GLP-1RAs reduce caloric intake and result in 1–4 kg weight loss on average over several months. The long-term weight loss effect of GLP-1RAs has also been validated in a recent large-scale clinical trial, which reported a weight loss up to 20.9% after 72-week treatment of tirzepatide [22]. Sustained treatment with semaglutide leads to a reduction of blood pressure by 1.8 to 4.6 mm Hg compared with other glucose-lowering agents or placebo [82, 83]. Modest reductions in LDL-cholesterol, total cholesterol, and triglycerides are also observed in patients receiving GLP-1RAs when compared with other antidiabetic agents [84].

GLP-1 and its analogues have been shown to possess direct effects on improving cardiomyocyte viability, cardiac function, and vasodilation. DPP-4i and GLP-1RAs are able to enhance vasodilation by increasing nitric oxide production, promote myocyte glucose uptake, and increase coronary flow, thus providing cardioprotective effects during the acute phase of ischemic heart diseases [85–88]. Therefore, direct protection of cardiovascular system and favorable effects on multiple cardiovascular risk factors (hyperglycemia, blood pressure, dyslipidemia, body weight) observed in patients receiving GLP-1RAs may provide an explanation for the cardiovascular benefits of GLP-1RAs (Fig. 2).

## HF

The prevalence of HF has become an increasing concern for patients with T2DM. Large CVOTs and other smaller studies have shown an association between the use of GLP-1RAs and DPP-4i on the relative rates of HF hospitalization.

### Effects of GLP-1RAs on HF

On the whole, meta-analyses have shown that GLP-1RAs reduced the risk of developing HF (*HR* = 0.62, 95% CI 0.31–1.02) [89]. Individual agents within this class did show some variability, however. For example, liraglutide was associated with a non-significant increased risk of HF relative to placebo [odds ratio (*OR*) = 4.85, 95% CI 0.75–31.36] [89].

For patients with established HF and a reduced left ventricular ejection fraction (LVEF), the LIVE and FIGHT trials studied the effect of 2-year liraglutide use [90, 91]. Both studies reported almost no change in LVEF between the placebo and treatment groups (Table 3). Additionally, data was collected from ELIXA, LEADER, EXSCEL, SUSTAIN-6, HARMONY, PIONEER 6, REWIND, and AMPLITUDE-O [53, 54, 56, 59–63]. All supported a non-significant difference in rates of hospitalization for HF relative to the placebo (Table 1).

**Table 3:** Trials of GLP-1RAs on HF outcomes

Characteristic	LIVE (2016) [91]	FIGHT (2016) [90]
Drug	Liraglutide	Liraglutide
Comparative agent	Placebo	Placebo
Follow-up period (weeks)	24.0	25.7
Inclusion criteria	Patients aged 30–85 years with CHF; LVEF ≤ 45%; functional class I–III; patients both with and without T2DM were included	HF with LVEF ≤ 40%; hospitalization for acute HF within last 14 d despite prior treatment; preadmission dose of 40 mg of furosemide or equivalent
Exclusion criteria	Class IV HF; MI within the last 3 months; type 1 diabetes; HbA1c > 10%; heart disease hospitalization in last 30 d; Afib with ventricular frequency above 100/min at rest; coronary revascularization within the last 3 months; obstructive hypertrophic cardiomyopathy; use of GLP-1RAs within last 30 d	Acute coronary syndrome or intervention; intolerance to GLP-1RAs; severe renal, hepatic, or pulmonary failure
Primary endpoint	Change in LVEF	Global rank score (higher values indicated better health): time to death, time to rehospitalization for HF, and time-averaged proportional change in N-terminal pro-B-type natriuretic peptide level from baseline to 180 d
Results of endpoint	Absolute increase in LVEF: (0.8 ± 4.7)% vs. (1.7 ± 4.4)% [mean difference − 0.9% (95% CI − 2.1–0.3), *P* = 0.15]	Death: 12% vs. 11% (*HR* = 1.10, 95% CI 0.57–2.14; *P* = 0.78); rehospitalization for HF: 41% vs. 31% (*HR* = 1.30, 95% CI 0.89–1.88; *P* = 0.17); composite of death or rehospitalization for HF: 47% vs. 39% (*HR* = 1.30, 95% CI 0.92–1.83; *P* = 0.14)

*CHF* congestive heart failure, *LVEF* left ventricular ejection fraction, *HbA1c* glycated hemoglobin, *HF* heart failure, *T2DM* type 2 diabetes mellitus, *MI* myocardial infarction, *Afib* atrial fibrillation, *GLP-1RAs* GLP-1 receptor agonists, *HR* hazard ratio, *CI* confidence interval

However, Arturi et al. [92] studied a similar patient population and found improved LVEF after a 52-week treatment with liraglutide, and Chen et al. [93] found that in patients who had HF and a preserved LVEF, a notable increase in LVEF with a 1-week treatment of liraglutide. The patients studied by Chen et al. had an additional history of STEMI + percutaneous coronary intervention (PCI) or non-STEMI. Similar to this, in a meta-analysis study including 6 randomized controlled trials, Huang et al. [94] found that patients who were treated with GLP-1RAs after a heart attack related PCI, showed improved LVEF and reduced infarct size. Though these findings were heterogeneous, there was no added benefit of liraglutide and its use therefore in clinical practice for this given population is not supported [95]. Lastly, it is important to look at how other anti-diabetic therapies affect HF to select an appropriate drug regimen for patients with excessive risk factors or established HF. The EMPA-REG OUTCOMES trial showed that empagliflozin, a SGLT-2i, markedly reduced the risk for HF hospitalization relative to the placebo (*HR* = 0.65, 95% CI 0.50–0.85, *P* = 0.002) [96]. Though both liraglutide and empagliflozin reduce blood pressure, a risk factor to HF, the difference in HF hospitalization cannot be accounted for even when blood pressure is pre-controlled, and Scheen et al. [89] have postulated that this discrepancy in outcomes may be rooted in the diuretic function of SGLT-2i.

### Effects of DPP-4i on HF

DPP-4 has been shown to directly increase factors that are predominant in HF. For example, DPP-4 cleaves BNP (1–32) into BNP (3–32), which is regularly detected in the plasma of patients with congestive HF [97].

Lourenço et al. [98, 99] sought to uncover an association between DPP-4 levels and the risk of mortality in patients who had HF with a reduced ejection fraction. There was an observed U-shape association between serum DPP-4 and mortality in patients with chronic systolic HF, and Lourenço et al. [98, 99] advised that DPP-4i will only be beneficial in instances where serum DPP-4 is extremely upregulated, 625 ng/ml or higher. Clinically, these findings should be considered when treating patients with DPP-4i, especially in those patients with HF.

Results from EXAMINE, SAVOR-TIMI53, and TECOS also shed some light on HF. EXAMINE showed a slight, but non-significant decrease in HF related hospitalization, while SAVOR-TIMI53 showed an increase in HF hospitalization with DPP-4i use. The TECOS trial showed no difference between the placebo and a sitagliptin group (Table 2) [47–49]. Pooled data from these three trials showed that rates of HF hospitalization were not significantly different between DPP-4i and placebo, suggesting that the effect may not be a class effect [100, 101]. Another meta-analysis including 43 trials (*n* = 68,775) and 12 observational studies (*n* = 1,777,358) concluded that the relative effect of DPP-4i as a class on HF in T2DM patients is uncertain. Recently completed CARMELINA and CAROLINA trials also suggested that linagliptin did not increase HF hospitalization compared to placebo or glimepiride [57, 58]. However, DPP-4i should be used with caution in T2DM patients with existing HF or risk factors [102].

## Conclusions

The development of incretin-based treatments has helped improve the quality of life and the management of symptoms for patients inflicted with T2DM. Both GLP-1RAs and DPP-4i have proven to be effective in restoring overall glycemic control, lowering HbA1c and lipid levels, amongst other benefits. Cardiovascular safety of antidiabetic medications has received increasing attention since 2008 and here we summarize the recent updates from both clinical trials and preclinical experiments on the cardiovascular outcomes of this class of glucose-lowering drugs. Randomized controlled trials before the issue of CVOTs by the FDA in 2008 mainly focused on the efficacy of the blood glucose-lowering effects and a broad spectrum of adverse effects, with limited numbers of cardiovascular adverse events and relatively short follow-up duration. Thus, these trials are not thoroughly discussed in this review. Although some in-class heterogeneity has been observed, these CVOTs have shown that DPP-4i and GLP-RAs are safe relative to major cardiovascular outcomes. In addition, LEADER, SUSTAIN-6, HARMONY, PIONEER 6, REWIND, and AMPLITUDE-O found that GLP-1RAs had cardioprotective effects independent of their ability to lower blood glucose levels. The picture remains incomplete, however, when assessing the mechanism by which this is achieved and if these cardiovascular effects can be generalized for the entire class of drugs. With regards to specific outcomes such as HF, there has been heterogeneity in the results with both GLP-1RAs and DPP-4i. There have also been varying outcomes within classes depending on the etiology of the patient’s HF. Therefore, more clinical trials utilizing a large generalizable T2DM patient population with less influence from baseline characteristics and longer follow-up durations are needed to help guide clinical decision-making and reduce the burden of T2DM while also promoting cardiovascular health.

## Data Availability

Not applicable.

## Abbreviations

ADA: Adenosine deaminase
ADVANCE: Action in Diabetes and Vascular Disease: Preterax and Diamicron MR Controlled Evaluation
Afib: Atrial fibrillation
BNP: Brain natriuretic peptide
cAMP: Cyclic adenosine 3′,5′-monophosphate
CI: Confidence interval
CKD: Chronic kidney disease
CVD: Cardiovascular disease
CVOTs: Cardiovascular outcome trials
DPP-4: Dipeptidyl peptidase-4
DPP-4i: DPP-4 inhibitor
eGFR: Estimated glomerular filtration rate
FDA: Food and Drug Administration
GAS: Interferon-gamma-activated sequence
GIP: Glucose-dependent insulinotropic polypeptide
GLP-1: Glucagon-like peptide-1
GLP-1R: GLP-1 receptor
GLP-1RAs: Glucagon-like peptide-1 receptor agonists
HbA1c: Glycated hemoglobin
HDL: High-density lipoprotein
HF: Heart failure
HNF-1α: Hepatocyte nuclear factor-1α
HR: Hazard ratio
hs-CRP: High-sensitivity C-reactive protein
IL-12: Interleukin-12
IVUS: Intravascular ultrasound
LDL: Low-density lipoprotein
LVEF: Left ventricular ejection fraction
MACE: Major adverse cardiovascular events
MI: Myocardial infarction
MMPs: Matrix metalloproteases
PAI-1: Plasminogen activator inhibitor-1
PCI: Percutaneous coronary intervention
PKA: Protein kinase A
PWV: Pulse wave velocity
SGLT-2i: Sodium/glucose cotransporter-2 inhibitors
STAT1α: Signal transducer and activator of transcription 1α
T1DM: Type 1 diabetes mellitus
T2DM: Type 2 diabetes mellitus
TGF-β: Transforming growth factor-β
TLR4: Toll-like receptor 4
TNF-α: Tumor necrosis factor-α
TRIF: TIR-domain-containing adapter-inducing interferon-β
UKPDS: United Kingdom Prospective Diabetes Study
VLDL: Very low-density lipoprotein
